# SRV2 promotes mitochondrial fission and Mst1-Drp1 signaling in LPS-induced septic cardiomyopathy

**DOI:** 10.18632/aging.102691

**Published:** 2020-01-17

**Authors:** Xiuling Shang, Yingrui Zhang, Jingqing Xu, Min Li, Xiaoting Wang, Rongguo Yu

**Affiliations:** 1 Department of Critical Care Medicine, Fujian Provincial Hospital, Fujian Provincial Center for Critical Care Medicine, Fujian Medical University, Fuzhou, Fujian 350001, China; 2 Department of Critical Care Medicine, Peking Union Medical College Hospital, Peking Union Medical College, Chinese Academy of Medical Science, Beijing 100730, China

**Keywords:** SRV2, septic cardiomyopathy, mitochondrial fission, Mst1-Drp1 axis

## Abstract

Mitochondrial fission is associated with cardiomyocyte death and myocardial depression, and suppressor of ras val-2 (SRV2) is a newly discovered pro-fission protein. In this study, we examined the mechanisms of SRV2-mediated mitochondrial fission in septic cardiomyopathy. Western blotting, ELISA, and immunofluorescence were used to evaluate mitochondrial function, oxidative balance, energy metabolism and caspase-related death, and siRNA and adenoviruses were used to perform loss- and gain-of-function assays. Our results demonstrated that increased SRV2 expression promotes, while SRV2 knockdown attenuates, cardiomyocyte death in LPS-induced septic cardiomyopathy. Mechanistically, SRV2 activation promoted mitochondrial fission and physiological abnormalities by upregulating oxidative injury, ATP depletion, and caspase-9-related apoptosis. Our results also demonstrated that SRV2 promotes mitochondrial fission via a Mst1-Drp1 axis. SRV2 knockdown decreased Mst1 and Drp1 levels, while Mst1 overexpression abolished the mitochondrial protection and cardiomyocyte survival-promoting effects of SRV2 knockdown. SRV2 is thus a key novel promotor of mitochondrial fission and Mst1-Drp1 axis activity in septic cardiomyopathy.

## INTRODUCTION

Sepsis-induced cardiomyopathy, which is characterized by left ventricular dilation and decreased ejection fraction, significantly increases perioperative mortality [1]. However, there are few effective drugs and therapeutic approaches for patients with septic cardiomyopathy. Identification of the molecular mechanisms underlying the development of septic cardiomyopathy might help identify new therapeutic targets and improve the efficacy of septic cardiomyopathy treatments as well as its prognosis [2].

Mitochondria are primarily responsible for ATP generation in cardiac cells. Structurally, mitochondria are highly plastic organelles that undergo continuous fusion, fission, trafficking, and mitophagy [3]. Mitochondrial homeostasis is controlled by mitochondrial fission. For example, active mitochondrial fission has been associated with cardiomyocyte death in a myocardial reperfusion model [4, 5]. During oxidative stress, endothelial cell survival rates are also impacted by fission-initiated, caspase-9-related apoptosis [6]. Moreover, fission also facilitates high-fat-mediated hepatic injury [7, 8] and diabetic nephropathy. Therefore, fission may be a critical regulator of mitochondrial function as well as cell survival [9]. This has been observed in various cancers, such as gastric cancer, liver tumors, and thyroid carcinoma [10]. However, few studies have explored the downstream effects and key inducers of mitochondrial fission in septic cardiomyopathy.

Suppressor of ras val-2 (SRV2), a newly discovered pro-fission protein, affects mitochondrial shape and activates mitochondrial fission via multiple mechanisms [11]. First, SRV2 can promote interactions between Drp1 and mitochondria [12]. Subsequently, SRV2 promotes oligomerization of Drp1, which then forms a ring around mitochondria that constricts and cuts them into several fragments. SRV2 also increases the expression of stress fibers, such as F-actin [13], that facilitate Drp-1-mediated mitochondrial division [14]. In this study, we conducted several experiments to understand the effects of SRV2 on fission in septic cardiomyopathy.

Macrophage stimulating 1 (Mst1), a key factor in the Hippo signaling pathway, is important for mitochondrial structural maintenance and functional preservation [15]. For example, Mst1 activation is associated with mitochondrial stress in LPS-treated hepatocytes. In fatty liver disease, inhibition of Mst1 reduces mitochondrial autophagy [16]. Interestingly, mitochondrial membrane potential and apoptosis are also affected by Mst1 in hyperglycemia-treated retinal epithelial cells. In contrast, loss of Mst1 attenuates renal ischemia reperfusion injury by maintaining mitochondrial homeostasis [17]. In addition, Mst1 knockdown enhances cardiomyocyte viability by improving mitochondrial performance through mitochondrial autophagy. The effects of Mst1 on mitochondrial fission have been widely reported in many kinds of cancers, such as gastric, lung, pancreatic, liver, and colorectal cancer [18]. In the present study, we explored whether SRV2-related mitochondrial fission is mediated by Mst1 in, and whether it contributes to the pathogenesis of, septic cardiomyopathy.

## RESULTS

### SRV2 is upregulated in septic cardiomyocytes and correlates with cardiac dysfunction

**Figure 1:**
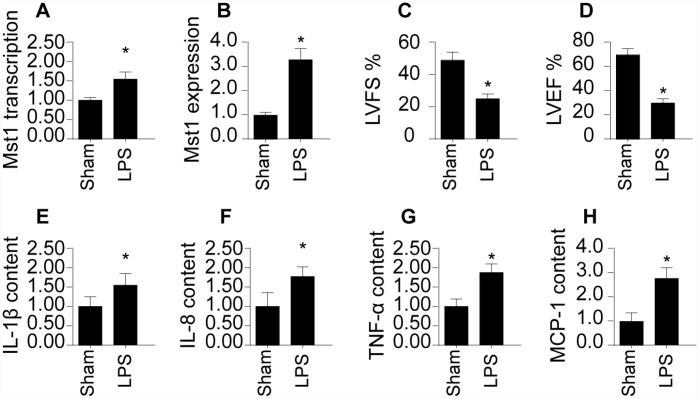
**SRV2 is upregulated in LPS-treated cardiomyocytes.** (**A**) RNA was isolated from LPS-treated heart tissues and qPCR was performed to analyze SRV2 transcript levels. (**B**) Protein was isolated from LPS-treated heart tissues, and Western blots were used to quantify SRV2 protein expression in cardiomyocytes. (**C**–**D**) Echocardiography was used to evaluate cardiac function after LPS injections. LVEF: left ventricular ejection fraction, LVFS: left ventricular fractional shortening. (**E**–**H**) Blood was collected after treatment and IL-1β, IL-8, TNF-α, and MCP-1 levels were determined using ELISA. *p<0.05 vs. control group.

First, we measured alterations in SRV2 levels via qPCR and Western blotting in a mouse model of septic cardiomyopathy. As shown in Figure 1A and 1B, compared to the sham group, SRV2 transcript and protein levels were significantly elevated in mice with LPS-induced septic cardiomyopathy. Echocardiography was used to examine associations between SRV2 upregulation and sepsis-related myocardial damage. As shown in Figure 1C and 1D, compared to the sham group, LVEF and LVFS were significantly reduced after LPS treatment, suggesting a loss of cardiac contractile function. In addition, inflammation factors such as IL-1β, IL-8, TNF-α, and MCP-1, were markedly increased in mice injected with LPS (Figure 1E–1H). Together, these results indicate that SRV2 is activated by LPS and is associated with heart failure in a mouse model of septic cardiomyopathy.

### Loss of SRV2 attenuates cell death and sustains cardiomyocyte function

**Figure 2:**
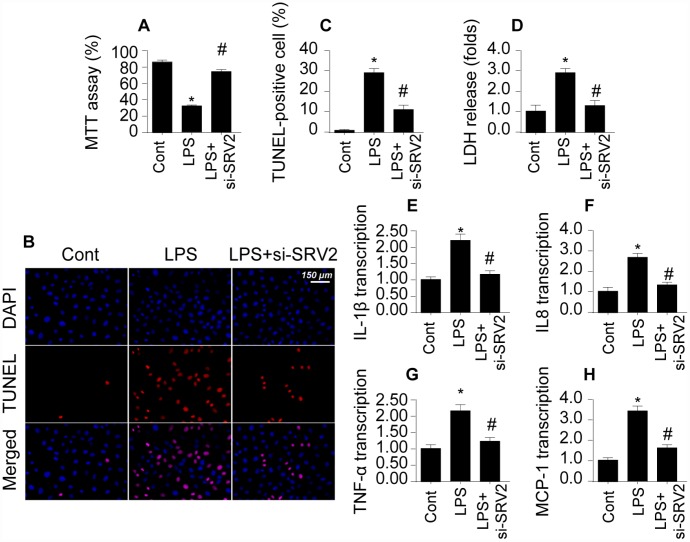
**Loss of SRV2 attenuates cardiomyocyte death and reduces inflammation response in LPS-induced septic cardiomyopathy.** (**A**) Cardiomyocyte viability was measured via MTT assay after transfection of siRNA against SRV2. (**B**–**C**) TUNEL staining was used to evaluate numbers of apoptotic cardiomyocytes after SRV2 siRNA transfection. (**D**) An LDH release assay was used to examine cardiomyocyte damage in response to LPS treatment and after SRV2 siRNA transfection cardiomyocytes. (**E**–**H**) RNA was isolated from LPS-treated cardiomyocytes and qPCR was performed to analyze IL-1β, IL-8, TNF-α, and MCP-1 transcript levels. *p<0.05 vs. control group, #p<0.05 vs. LPS group.

To determine whether SRV2 upregulation directly causes cardiac damage, a loss of function assay was performed by transfecting cardiomyocytes with siRNA against SRV2. Cardiomyocyte viability was then measured in an MTT assay. As shown in Figure 2A, compared to the control group, cardiomyocyte viability was reduced by LPS treatment; this effect was reversed by SRV2 siRNA transfection. Cardiomyocyte death was further analyzed with TUNEL staining and an LDH release assay. As shown in Figure 2B and 2C, compared to the control group, the number of apoptotic cells increased greatly after exposure to LPS treatment. SRV2 knockdown also reduced the ratio of apoptotic to normal cardiomyocytes. In accordance with these findings, LDH levels in the culture medium were markedly increased in response to LPS treatment and returned to normal levels after siRNA-induced silencing of SRV2 (Figure 2D). SRV2 knockdown also decreased the transcription of inflammatory factors. As shown in Figure 2E–2H, compared to the control group, LPS treatment upregulated the transcription of IL-1β, IL-8, and MCP-1 in cardiomyocytes, and inhibition of SRV2 reversed this effect. These results indicate that downregulation of SRV2 attenuates LPS-induced cardiomyocyte death and dysfunction.

### SRV2 activation is associated with mitochondrial fission

**Figure 3:**
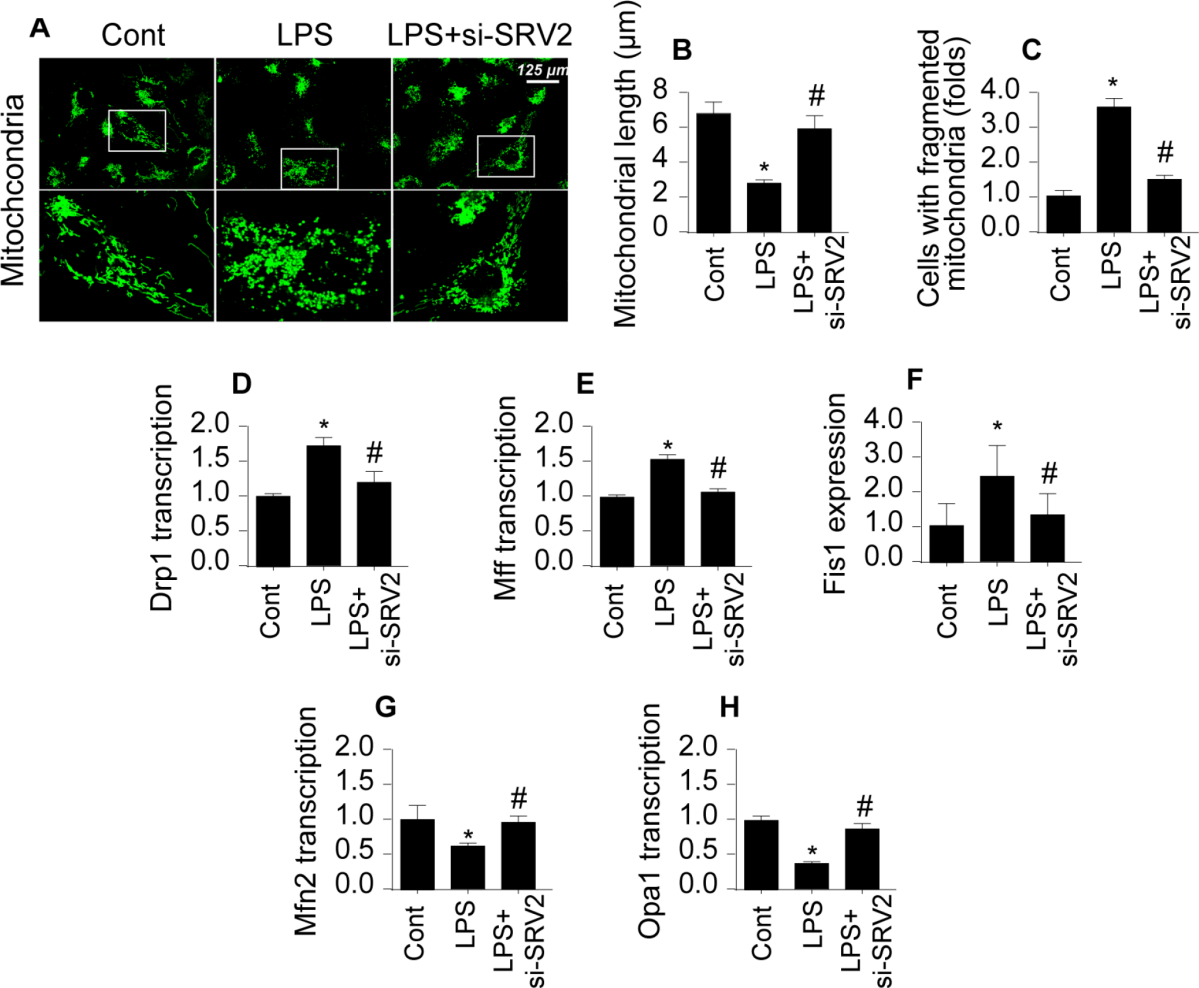
**SRV2 triggers mitochondrial fission in cardiomyocytes.** (**A**–**C**) Average mitochondrial length was measured and proportion of fragmented mitochondria was evaluated in an immunofluorescence assay after cardiomyocytes were transfected with siRNA against SRV2. (**D**–**F**) RNA was isolated from LPS-treated cardiomyocytes and qPCR was performed to analyze Drp1, Fis1, and Mff transcript levels. (**G**–**H**) RNA was isolated from LPS-treated cardiomyocytes and qPCR was performed to analyze Mfn2 and Opa1 transcript levels. *p<0.05 vs. control group, #p<0.05 vs. LPS group.

Next, we examined mitochondrial fission, an early indicator of cardiomyocyte damage [19, 20], to understand the molecular mechanism by which SRV2 decreases cardiomyocyte functions and survival in LPS-mediated septic cardiomyopathy. First, an immunofluorescence assay was performed to quantify mitochondrial fission. As shown in Figure 3A–3C, compared to the control group, mitochondrial fission was activated by LPS in cardiomyocytes, as evidenced by decreased mitochondrial length and increased mitochondrial fragmentation. Interestingly, SRV2 knockdown inhibited LPS-mediated mitochondrial fission, as indicated by reversal of mitochondrial network alterations and increased mitochondrial length (Figure 3A–3C). In addition, transcription of mitochondrial fission-related proteins, including Drp1, Fis1, and Mff, increased rapidly after exposure to LPS (Figure 3D–3F). Furthermore, levels of anti-fission factors such as Mfn2 and Opa1 markedly decreased after LPS treatment (Figure 3G–3H). These data suggest that LPS stress triggers mitochondrial fission. In contrast, Drp1, Fis1, and Mff levels decreased (Figure 3D–3F), while Mfn2 and Opa1 levels increased (Figure 3G and 3H), after deletion of SRV2 in LPS-treated cardiomyocytes. These results indicate that LPS-mediated upregulation of SRV2 promotes mitochondrial fission.

### Inhibition of SRV2-mediated mitochondrial fission promotes cell survival and sustains cardiomyocyte function

**Figure 4:**
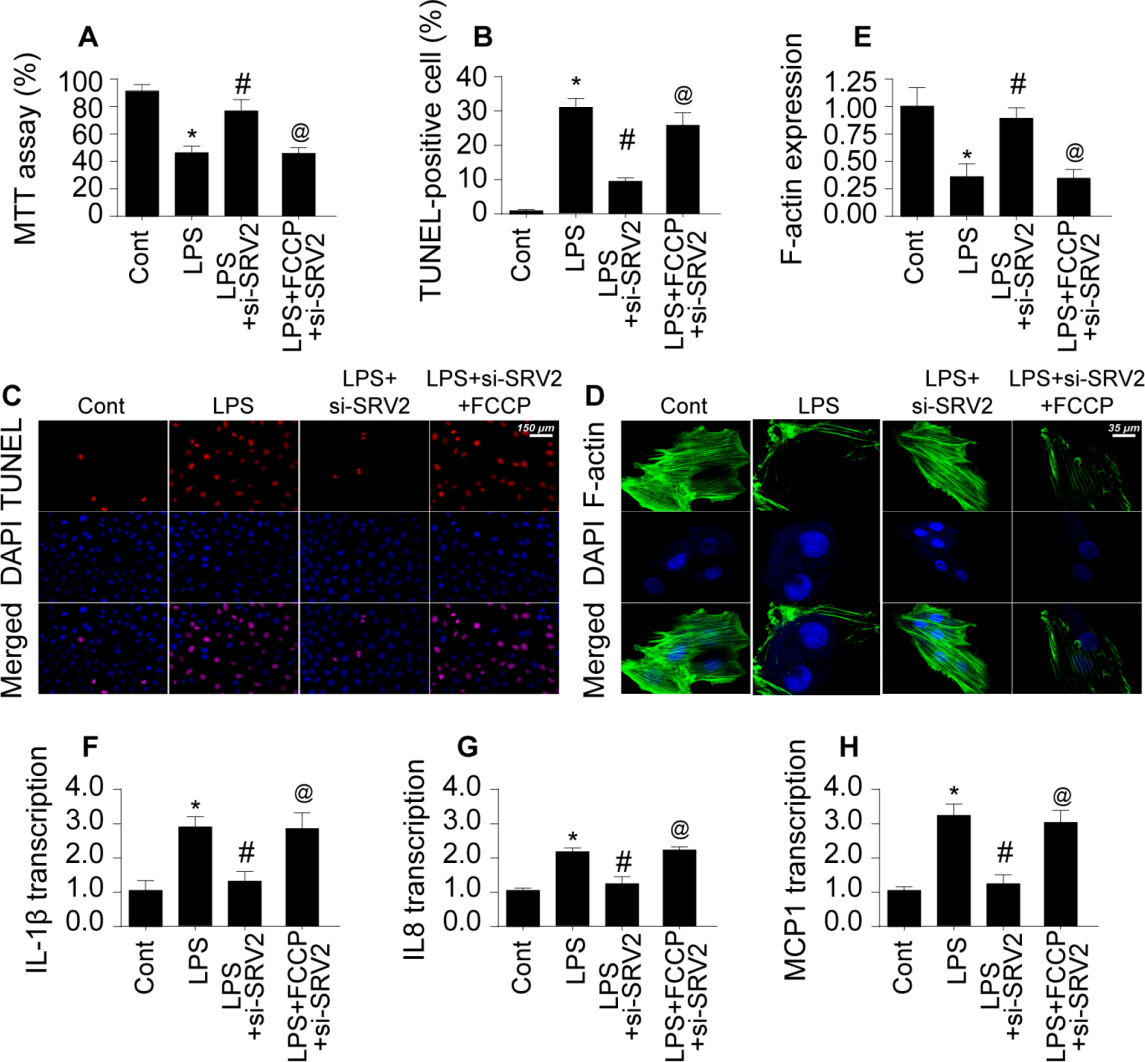
**Re-activation of mitochondrial fission reverses the pro-survival effects of SRV2 knockdown in cardiomyocytes.** (**A**) Cardiomyocyte viability was measured via MTT assay after transfection of siRNA against SRV2 and addition of FCCP to the culture medium. (**B**–**C**) TUNEL staining was used to quantify numbers of apoptotic cardiomyocytes after SRV2 siRNA transfection and FCCP treatment. (**D**–**E**) Relative expression of F-actin in cardiomyocytes was measured in an immunofluorescence assay after SRV2 siRNA transfection and FCCP treatment. (**F**–**H**) RNA was isolated from LPS-treated cardiomyocytes and qPCR was performed to analyze IL-1β, IL-8, and MCP-1 transcript levels after SRV2 siRNA transfection and FCCP treatment. *p<0.05 vs. control group, #p<0.05 vs. LPS group, @p<0.05 vs. LPS+si-SRV2 group.

Next, we explored whether SRV2 induced cardiomyocyte damage through mitochondrial fission by measuring viability in SRV-2 knockdown cardiomyocytes treated with FCCP, an agonist of mitochondrial fission [21]. As shown in Figure 4A, LPS-induced cardiomyocyte damage was reversed by SRV2 knockdown, and FCCP treatment blocked this effect. In addition, although cardiomyocyte death as indicated by TUNEL staining (Figure 4B–4C) was attenuated by SRV2 knockdown after LPS treatment, FCCP increased the proportion of apoptotic cardiomyocytes.

In addition to cardiomyocyte death, we also examined structural alterations in the cardiomyocyte cytoskeleton, which is vital for cellular contraction [22]. Interestingly, expression of the cytoskeleton protein F-actin decreased after exposure to LPS, and SRV2 knockdown reversed this effect (Figure 4D–4E). FCCP again blocked the effects of SRV2 knockdown after FPS treatment. Furthermore, SRV2 knockdown reduced LPS-induced increases in inflammatory response as indicated by IL-1β, IL-8, and MCP-1 transcription (Figure 4F–4H) to near-normal levels in cardiomyocytes, and FCCP treatment again blocked this effect (Figure 4F–4H). Together, these results indicate that inhibition of SRV2 protects cardiomyocytes against LPS-induced stress by inhibiting mitochondrial fission.

### SRV2-induced mitochondrial fission promotes mitochondrial damage

**Figure 5:**
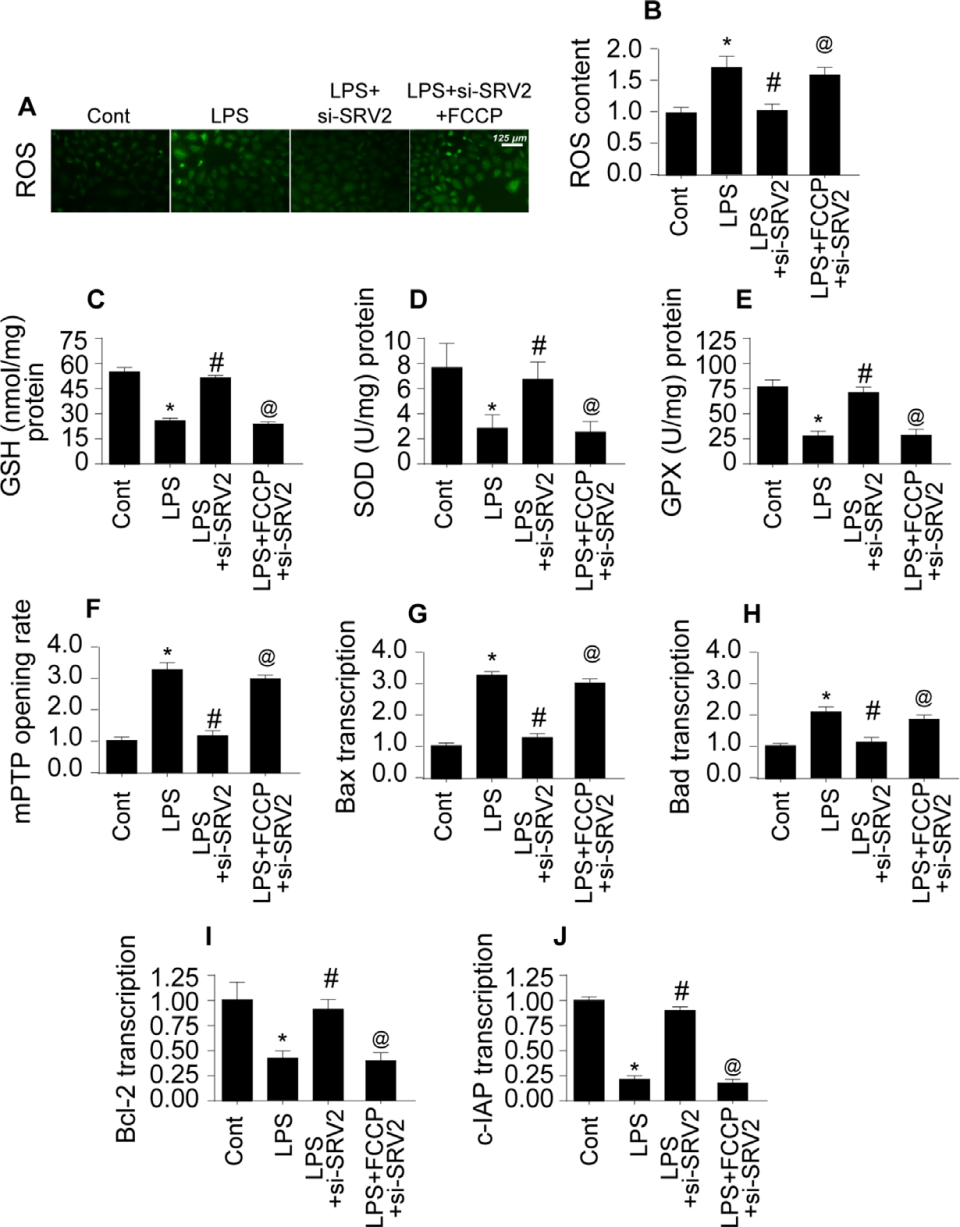
**SRV2-induced mitochondrial fission promotes mitochondrial damage.** (**A**–**B**) An ROS probe was used to detect ROS production in cardiomyocytes after transfection of siRNA against SRV2 and addition of FCCP to the culture medium. (**C**–**E**) SOD, GSH, and GPX levels were measured via ELISA in cardiomyocytes after SRV2 siRNA transfection and FCCP treatment. (**F**) mPTP opening was determined via ELISA in cardiomyocytes after SRV2 siRNA transfection and FCCP treatment. (**G**–**J**) After treatment, RNA was isolated from LPS-treated cardiomyocytes and qPCR was performed to analyze Bcl2, Bad, Bax, and c-IAP transcript levels after SRV2 siRNA transfection and FCCP treatment. *p<0.05 vs. control group, #p<0.05 vs. LPS group, @p<0.05 vs. LPS+si-SRV2 group.

To further characterize the molecular mechanism by which SRV-mediated mitochondrial fission promotes cardiomyocyte death, mitochondrial function and damage were measured [23]. Reactive oxygen species (ROS) are generated primarily by mitochondria, and excessive ROS production is a risk factor for myocardial depression [24]. Using an ROS probe, we found that ROS levels increased markedly in LPS-treated cardiomyocytes (Figure 5A–5B). SRV2 knockdown reduced ROS levels by inhibiting mitochondrial fission; FCCP restored increased ROS levels in SRV2-knockdown cardiomyocytes (Figure 5A–5B). Antioxidant levels increased after SRV2 knockdown (Figure 5C–5E), and decreased after subsequent FCCP treatment, in LPS-treated cardiomyocytes, suggesting that SRV2 inhibition exerts antioxidative effects in cardiomyocytes by inhibiting mitochondrial fission.

Mitochondrial damage is also characterized by the opening of mitochondrial permeability transition pores (mPTP) [25]. As shown in Figure 5F, compared to the control group, LPS increased the proportion of cardiomyocytes with open mPTPs. SRV2 knockdown prevented LPS-mediated mPTP opening, and FCCP treatment reversed this effect (Figure 5F). Opening of mPTPs resulted in increased transcription of pro-apoptotic mitochondrial genes after LPS treatment (Figure 5G–5J), and this effect was reversed by SRV2 knockdown-mediated inhibition of mitochondrial fission (Figure 5G–5J). Together, these results demonstrate that SRV2-mediated mitochondrial fission promotes mitochondrial damage, which in turn leads to cardiomyocyte dysfunction and death.

### Cardiomyocyte mitochondrial metabolism is disrupted by SRV2-induced mitochondrial fission

**Figure 6:**
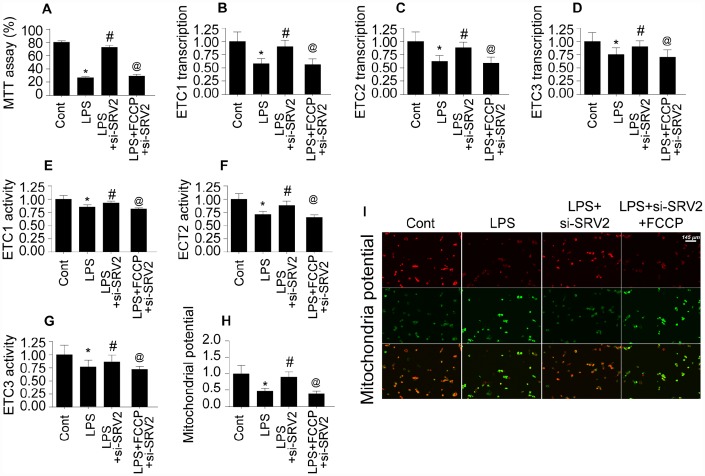
**Cardiomyocyte mitochondrial metabolism is disrupted by SRV2-induced mitochondrial fission.** (**A**) ATP production was measured in cardiomyocytes after transfection of siRNA against SRV2 and addition of FCCP to the culture medium. (**B**–**D**) RNA was isolated from LPS-treated cardiomyocytes and qPCR was performed to analyze ETC-1/2/3 transcript levels after SRV2 siRNA transfection and FCCP treatment. (**E**–**G**) Cardiomyocyte ETC-1/2/3 activities were measured via ELISA after SRV2 siRNA transfection and FCCP treatment. (**H**–**I**) The JC-1 probe was used to evaluate mitochondrial membrane potential in cardiomyocytes after SRV2 siRNA transfection and FCCP treatment; red-to-green fluorescence ratios indicate alterations in mitochondrial membrane potential. *p<0.05 vs. control group, #p<0.05 vs. LPS group, @p<0.05 vs. LPS+si-SRV2 group.

Mitochondrial energy metabolism is vital for cardiomyocyte survival and contraction [26]. ATP depletion and bioenergetic impairment have been observed in cardiomyocytes during septic cardiomyopathy [27]. Here, we investigated whether SRV2-mediated mitochondrial fission is also involved in cardiomyocyte metabolism dysregulation. As shown in Figure 6A, compared to the control group, ATP generation was decreased in LPS-treated cardiomyocytes. SRV2 knockdown increased ATP production, and FCCP attenuated this effect (Figure 6A). ATP is generated primarily at the mitochondrial electron transport chain (ETC) complex. Transcription of the ETC is reduced by LPS and restored to control levels by SRV2 knockdown (Figure 6B–6D). FCCP-induced reactivation of mitochondrial fission decreased ETC transcription (Figure 6B–6D). LPS, SRV2 knockdown, and FCCP also had similar effects on mitochondrial ETC activity (Figure 6E–6G). Together, these findings indicate that SRV2-mediated mitochondrial fission leads to ETC dysfunction. As a result of this ETC dysfunction, mitochondrial membrane potential was reduced, as evidenced by increased green JC-1 fluorescence in LPS-treated cardiomyocytes (Figure 6H–6I). SRV2 knockdown reversed this decrease in mitochondrial membrane potential, and FCCP-induced mitochondrial fission again decreased mitochondrial potential (Figure 6H–6I). Taken together, these results demonstrate that SRV2 knockdown, and the resulting suppression of mitochondrial fission, prevents LPS-induced dysregulation of cardiomyocyte energy metabolism.

### SRV2 promotes mitochondrial fission via the Mst1-Drp1 signaling pathway

**Figure 7:**
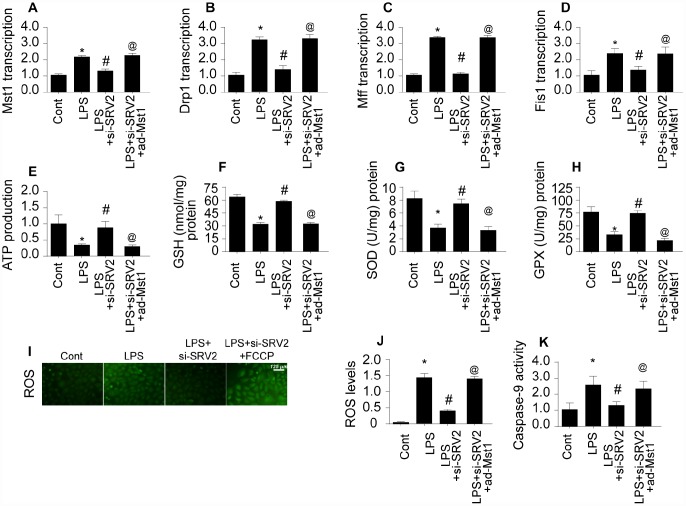
**SRV2 promotes mitochondrial fission by activating the Mst1-Drp1 signaling pathway.** (**A**–**B**) RNA was isolated from LPS-treated cardiomyocytes and qPCR was performed to analyze Mst1 and Drp1 transcript levels after transfection of Mst1 overexpression adenovirus following SRV2-knockdown. (**C**–**D**) Cardiomyocyte Mff and Fis1 transcript levels were measured through qPCR after Mst1 overexpression and SRV2 knockdown. (**E**) ATP production was measured in cardiomyocytes after Mst1 overexpression and SRV2 knockdown. (**F**–**H**) Cardiomyocyte SOD, GSH, and GPX levels were measured via ELISA after transfection of SRV2 siRNA and Mst1 overexpression adenovirus. (**I**–**J**) ROS production was measured using ROS probe (**K**) and caspase-9 activity was determined via ELISA in cardiomyocytes after transfection of SRV2 siRNA and Mst1 overexpression adenovirus. *p<0.05 vs. control group, #p<0.05 vs. LPS group, @p<0.05 vs. LPS+si-SRV2 group.

Lastly, we examined the signal transduction mechanism by which SRV2 promotes mitochondrial fission in LPS-treated cardiomyocytes [28]. Previous studies have reported that mitochondrial fission is primarily regulated by Drp1, which is the downstream effector of the Mst1 pathway [29, 30]. Mst1-induced mitochondrial apoptosis has also been identified as an important mechanism of mitochondrial damage. We therefore investigated whether SRV2 induced mitochondrial fission through the Mst1-Drp1 signaling pathway. Drp1 and Mst1 transcription increased rapidly in response to LPS treatment (Figure 7A–7B), and SRV2 knockdown prevented this upregulation (Figure 7A–7B). To determine whether the Mst1-Drp1 pathway is required for SRV2-induced mitochondrial fission, mitochondrial fission was measured after adenovirus expressing Mst1 was transfected into SRV2 knockdown cardiomyocytes. SRV2 knockdown again inhibited LPS-mediated mitochondrial fission, and Mst1 overexpression reversed this effect (Figure 7A–7B). Drp1, Mff, and Fis1 transcription were also upregulated in response to Mst1 overexpression in SRV2 knockdown cardiomyocytes (Figure 7C–7D). These data indicate that mitochondrial fission is re-activated by Mst1 overexpression in SRV2-knockdown cells.

The SRV2 knockdown-induced increase in ATP generation was also abolished by Mst1 overexpression (Figure 7E). In addition, antioxidant levels in SRV2 knockdown cardiomyocytes were similar to controls, and Mst1 overexpression decreased SOD, GSH, and GPX levels (Figure 7F–7H). ROS content was also increased by Mst1 overexpression in SRV2 knockdown cells (Figure 7I–7J). Finally, caspase-9 activity increased after LPS treatment, and this effect was reversed by SRV2 knockdown. However, Mst1 overexpression increased caspase-9 activity in SRV2 knockdown cardiomyocytes (Figure 7K). Taken together, these data indicate that SRV2 promotes mitochondrial fission by activating the Mst1-Drp1 signaling pathway.

## DISCUSSION

Septic cardiomyopathy is a transient left ventricular dysfunction triggered by excessive inflammatory response. Although numerous theories have been developed to explain the pathogenesis of septic cardiomyopathy, the most common cause is disruption of mitochondrial structure and function [31]. Several studies have demonstrated that protection of mitochondria can attenuate decreases in myocardial activity during septic cardiomyopathy [32]. Excessive mitochondrial damage is characterized by oxidative stress and energy metabolism disorders that inhibit contraction and promote death in cardiomyocytes [33]. Although many studies have explored the pathological role of mitochondria in septic cardiomyopathy [34], the upstream mediators of inflammation-induced mitochondrial damage have not yet been identified [35].

**Figure 8:**
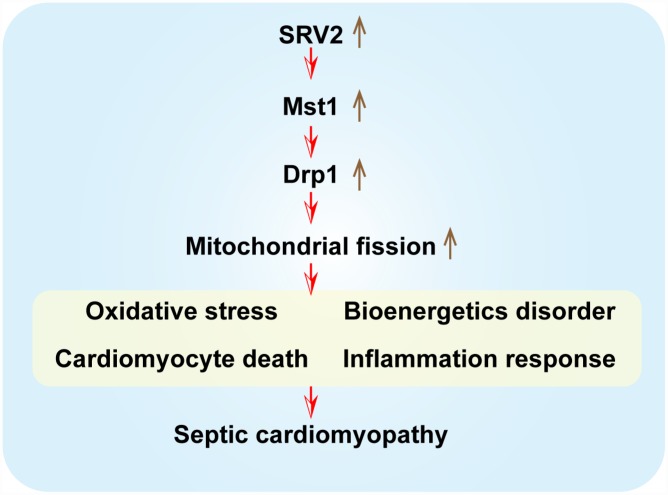
**A summary diagram of our results. SRV2 is upregulated in response to LPS-induced septic cardiomyopathy.** Excessive SRV2 upregulates Mst1 and Drp1, which in turn activate mitochondrial fission. Excessive fission induces cardiomyocyte death by promoting mitochondrial oxidative stress and inflammatory response and disrupting energy metabolism.

In this study, mice received LPS injections to induce septic cardiomyopathy. Our experimental results confirmed that inflammation-induced myocardial damage increased the expression of SRV2, a novel regulator of mitochondrial structure. Additionally, increased cardiomyocyte death and decreased cardiac function were associated with elevated SRV2 expression in cardiomyocytes [36, 37]. Loss of function assays were performed to further investigate the role of SRV2 in sepsis-related cardiac damage. Interestingly, SRV2 knockdown promoted cardiomyocyte survival and attenuated LPS-induced inflammatory response, confirming that SRV2 is a novel promoter of myocardial damage in septic cardiomyopathy (Figure 8). Previous studies have reported that SRV2 is involved in mitochondrial damage [38]. Here, SRV2 activated mitochondrial fission, which in turn promoted mitochondrial-associated cardiomyocyte apoptosis as evidenced by mitochondrial membrane potential loss, mitochondrial ROS overloading, antioxidant system suppression, cellular ATP depletion, pro-apoptotic factor release, and caspase family activation [39].

Furthermore, we found that SRV2 affects mitochondrial fission via the Mst1-Drp1 signaling pathway [40]. Overexpression of Mst1 abolished SRV2 knockdown-induced increases in cardiomyocyte survival and mitochondrial protection [41, 42]. This suggests that the SRV1-Mst1-Drp1 signaling pathway is a novel regulator of cardiomyocyte viability and mitochondrial homeostasis in the context of septic cardiomyopathy [43].

At the molecular level, accumulation of Drp1 and F-actin assembly initiate mitochondrial fission [44]. Drp1 forms a ring structure that causes mitochondrial contraction, and F-actin provides an adhesive force that helps Drp1 to complete mitochondrial contraction [45, 46]. Notably, SRV2 promotes polarized actin cable assembly, facilitates actin turnover [47], and enhances F-actin synthesis. Moreover, Drp1 accumulation on the mitochondrial surface is also regulated by SRV2. In these ways, SRV2 plays crucial regulatory roles in mitochondrial fission [48]. Our findings in the septic cardiopathy model also support this conclusion. Drp1 expression was downregulated after SRV2 knockdown, and this was followed by decreases the levels of other mitochondrial fission-related factors, such as Mff and Fis1 [49, 50]. However, the mechanism by which SRV2 modulates these mitochondrial fission-related factors remains unknown [51]. Notably, we demonstrated that SRV2 regulated Drp1 expression via Mst1-Hippo signaling; re-activation of the Mst1-Hippo pathway abolished the inhibitory effects of SRV2 knockdown on Drp1 expression. The Mst1-Hippo pathway has also been identified as an upstream regulator of mitochondrial fission [52]. For example, Mst1 activates mitochondrial fission by upregulating Drp1 in renal ischemia-reperfusion injury. In postinfarction cardiac injury [53], Mst1 activation is associated with the initiation of mitochondrial fission via JNK-mediated posttranscriptional modification of Drp1. Moreover, in endometriosis, Drp1-related mitochondrial fission is also affected by Mst1 [54]. The Mst-Hippo pathway has also been characterized as a cancer-killing pathway in several kinds of cancer, such as pancreatic, liver, gastric, and colorectal cancer [55, 56]. In this study, we identified an important mechanism by which Mst1 promotes cardiomyocyte death and mitochondrial fission [57]. These findings improve our understanding of the roles that the Mst1-Hippo pathway and SRV2 play in acute cardiac injury.

Some limitations should be considered when interpreting the results of this study. First, the SRV2 knockdown assay was performed *in* *vitro*, and animal studies and human research are needed to verify our findings. Furthermore, although we found that SRV2 modulates Drp1 expression, it remains unknown whether the Mst1-Hippo pathway also regulates other mitochondrial fission-related factors.

## MATERIALS AND METHODS

### Animals

Eight-week old neonatal C57BL/6 mice (Oriental Bio Service Inc., Nanjing) were maintained in standard cages on a 12 h light/dark cycle at 22°C ± 2°C with 55–65% relative humidity and given food and water *ad libitum*. Animal care and experimental procedures were conducted in accordance with the guidelines established by the Institutional Animal Care and Use Committees at Fujian Medical University.

### Mouse model and drug administration

The septic cardiomyopathy mouse model was established as previously described with minor adjustments. Thirty C57BL/6 mice were randomly divided between the normal saline control group (n=10) and an LPS-induced group (n=20). Mice in the LPS-induced group were injected intraperitoneally with LPS (10 mg/kg) purchased from Sigma-Aldrich (St. Louis, MO). Mice in the normal saline control group were injected with an equal volume of sterile saline [58].

### Echocardiographic assessment

In order to evaluate left ventricular (LV) function in the mouse septic cardiomyopathy model, transthoracic echocardiography was performed according to previously described procedures [59], including left ventricular ejection fraction (LVEF) and left ventricular fractional shortening (LVFS) parameters. All analyses were performed by a single investigator who was blinded to the experimental groups [60].

### ELISA assay

Blood was collected from the mice through eyeball extraction 24 h after LPS injection, and serum was separated by centrifugation at 3500 rpm at 4°C for 15 min. An ELISA kit (Invitrogen, Carlsbad, CA, USA) was used to measure levels of inflammatory cytokines (IL-1β, IL-8, TNF-α, and MCP-1) according to the manufacturer’s protocols. Emission was assayed at 450 nm relative to a reference wavelength using a microplate reader (Bio-Rad, Hercules, CA, USA) [61].

### Reverse transcription-quantitative polymerase chain reaction (RT-qPCR)

Total RNA was extracted from left atrial (LA) tissues using TRIzol Reagent (Invitrogen, Carlsbad, CA), and single-stranded cDNA was transcribed using the PrimeScript™ RT reagent Kit with gDNA Eraser (Takara, Dalian, China). RT-qPCR was performed on an ABI Prism 7500 Sequence Detection system (Applied Biosystems; Thermo Fisher Scientific, Inc.). The thermocycling conditions were as follows: 50°C for 2 min, then 40 cycles of 95°C for 30 sec and 60°C for 1 min. Transcript levels were measured relative to GAPDH using a calibration curve [62].

### Western blot analysis

Total protein was isolated from samples with lysis buffer. Proteins of interest were separated on SDS-PAGE gels, transferred to PVDF membranes (Millipore, Hong Kong, China), and incubated with Mst1 primary antibody (1:1000, Cell Signaling Technology, #3682) followed by horseradish peroxidase (HRP)-conjugated secondary antibody. The protein bands were detected by chemiluminescence (ECL) and were visualized using a Kodak Image Station 4000 (Rochester, NY). Band densities were quantified using the Quantity One analysis system (Bio-Rad Laboratories, UK) [63].

### Immunohistochemistry and immunofluorescence staining

Sections were incubated with diluted Tom20 (1:1,000, Abcam, #ab186735) and then with fluorescent secondary antibodies. DAPI (1:1; Servicebio Technology, Wuhan, China) was used for nuclear visualization. Images were captured using an Olympus fluorescence microscope at 400× magnification [64].

### Cell culture and transfection

The rat embryonic ventricular cardiomyocyte H9C2 cells were purchased from American Type Culture Collection (ATCC). The cells were maintained in DMEM (Hyclone) supplemented with 10% fetal bovine serum (FBS; Hyclone), 100 U/mL penicillin (Sigma), and 100 μg/mL streptomycin (Sigma) at 37°C in a humidified atmosphere with 5% CO_2_ [65]. Prior to experiments, cells were grown to 80-90% confluence, then transfected for 24 h with siRNA against SRV2 and Mst1 adenovirus at a multiplicity of infection of 50, achieving a 90% transduction efficiency. Cells were then subjected to serum starvation (0.4% FBS) for 24 h and treated with LPS at 20 μΜ for 24 hours [66].

### Mitochondrial membrane potential assay

Mitochondrial membrane potential (MMP) was determined using a JC-1 probe (BD Biosciences, San Diego, CA, USA). Briefly, after incubation with 10 μg/mL JC-1 in the dark for 20 min at 37°C, the cells were washed with PBS and observed using a confocal microscope (Leica Microsystems, Heidelberg, Germany). MMP was quantified by measuring the 590/488 fluorescence intensity ratio [67].

### ATP concentration

Mitochondrial ATP concentration was measured using an ATP quantification kit according to manufacturer’s instruction (Invitrogen, USA) [62]. ATP concentrations were normalized to total protein levels [68].

### Measurement of oxidative factors

ROS levels were measured using the Fluorometric Intracellular ROS kit (Sigma-Aldrich; Merck KGaA) [69]. Levels of glutathione (GSH), superoxide dismutase (SOD), and glutathione peroxidase (GPX) levels were measured to quantify oxidative stress using commercial kits from Nanjing Jiancheng Bio-Technology Co., Ltd., Nanjing, China [70].

### TUNEL assay

Cell apoptosis was measured in a TUNEL assay using an Apoptosis In Situ Detection Kit (Abcam, Cambridge, MA, USA) according to the manufacturer’s instructions. A Leica TCS-SP laser scanning confocal microscope (Leica Microsystems, Heidelberg, Germany) was used to take photomicrographs [71].

### Statistical analysis

Statistical analysis was performed using SPSS 22.0 software. Continuous variables with normal distributions were tested using one-way ANOVAs followed by the Tukey post-test; values are expressed as means ± SEM. The Kruskal-Wallis test was used for non-normally distributed variables; values are expressed as medians and interquartile ranges. Differences between groups were analyzed by studentʼs independent t-tests or Mann–Whitney U tests [72]. AF incidence across groups was analyzed using the Fisher exact test; values are expressed as percentages. P-values < 0.05 were considered statistically significant.
